# INTENSITY, DURATION AND TYPE OF PHYSICAL ACTIVITY REQUIRED TO IMPROVE FUNCTION IN KNEE OSTEOARTHRITIS

**DOI:** 10.1590/1413-785220172501166212

**Published:** 2017

**Authors:** RICARDO AKIHIRO KIRIHARA, FELLIPE BRAVIM CATELAN, FABIANE ELIZE SABINO DE FARIAS, CLEIDNÉIA APARECIDA CLEMENTE DA SILVA, CLAUDIA HELENA DE AZEVEDO CERNIGOY, MÁRCIA UCHOA DE REZENDE

**Affiliations:** 1. Universidade de São Paulo, Faculdade de Medicina, Hospital das Clinicas, Instituto de Ortopedia e Traumatologia, São Paulo, Brazil.

**Keywords:** Osteoarthritis, Knee, Patient education as topic, Motor activity, Treatment outcome

## Abstract

**Objective:**

To evaluate the effects of physical activity intensity, type and duration in patients with knee osteoarthritis (KOA)***.***

**Methods:**

A retrospective study of 195 KOA patients who were followed for two years after receiving educational material about KOA with or without attending classes. The patients were evaluated at baseline and 24 months. At the evaluations, the patients answered questionnaires pertaining to pain and function (WOMAC, Lequesne, VAS and SF-36); reported the intensity, duration and type of exercise performed per week; and performed the Timed Up & Go (TUG) and Five Times Sit-to-Stand (FTSST) tests***.***

**Results:**

Increased age affected improvements in the TUG results (p=0.017). The type, intensity and duration of physical activity did not correlate with pain, function or quality of life improvements (p>0.05), but the TUG results were on average 4 seconds faster among the patients who practiced intense physical activity and/or exercised for more than 180 minutes per week and/or performed isolated weight training or swam compared with those who remained sedentary after 2 years (p=0.01; p<0.001; p=0.01; p=0.04, respectively)***.***

**Conclusions:**

Patients with KOA should aim for intense physical activity and/or more than 180 minutes of exercise per week and/or weight training (bodybuilding) for relevant pain reduction and functional improvement.***Level of Evidence II, Retrospective Study.***

## RESUMO

**Objetivo:**

***Avaliar o efeito da intensidade, tipo e tempo da atividade física semanal em pacientes com osteoartrite do joelho (OAJ) .***

**Métodos:**

***Cento e noventa e cinco pacientes portadores de OAJ foram acompanhados por dois anos após receberam material educacional sobre OAJ, com ou sem aulas. Os pacientes responderam aos questionários de dor, função, qualidade de vida (WOMAC, Lequesne, EVA e SF-36), intensidade, frequência e tipo de atividade física semanal realizada, além de realizaram os testes de senta e levanta (TSL) e "Timed-Up-and-Go" (TUG) no momento da inclusão e após 24 meses.***

**Resultados:**

***O aumento da idade dos pacientes acarretou menor chance de melhora no TUG (p=0,017). O tipo de atividade física, intensidade e frequência não mostraram correlação com melhoras algo-funcionais e de qualidade de vida (p>0,05), porém os resultados do TUG foram em média 4 seg mais rápidos em pacientes que praticavam atividade física intensa e/ou acima de 180 min por semana e/ou musculação isolada, ou ainda, musculação ou natação, em relação aos pacientes sedentários (p=0,01; p<0,001; p=0,01; p=0,04, respectivamente) após dois anos. Pacientes praticantes de musculação tinham menos dor que os sedentários após o programa (p=0,009) .***

**Conclusão:**

*Pacientes com OAJ são aconselhados a objetivar atividade física intensa e/ou acima de 180 min por semana e/ou musculação para atingir melhora algo-funcional relevante.* ***Nível de Evidência II, Estudo Retrospectivo.***

## INTRODUCTION

Osteoarthritis is the leading cause of disability among chronic diseases.^1^ ^-^ ^3^ The societal and personal costs of functional limitation resulting from osteoarthritis are high among older adults.^4^ ^,^ ^5^ Medical expenses among the elderly are more closely related to functional losses than to life expectancy.^6^ ^,^ ^7^

A lack of regular vigorous physical activity is a potentially modifiable risk factor that could substantially reduce functional decline and related health care costs. Prevention/intervention programs should include regular vigorous physical activity, weight maintenance, and medical intervention for health needs.^8^

We developed an educational program for patients with knee osteoarthritis (KOA) that led to functional improvement and increased adherence to regular physical activity.^9^ ^,^ ^10^ However, the intensity, duration and type of physical activity that is necessary to produce significant functional gains is still unknown. Based on this need, we searched for a correlation between pain, functional and quality of life improvements and absolute results and the intensity, duration and type of physical activity practiced by patients to determine a physical activity goal that would allow patients with KOA to obtain clinically relevant functional improvements.

## METHODS

This study was performed at the Department of Orthopedics and Traumatology, São Paulo, Brazil, after it was approved by the Ethics Committee for the Analysis of Research Projects (CAPPesq) under protocol number 0622/11. 

Clinical trials registration number: NCT01572051.

One hundred ninety-five patients with KOA (46 men, 149 women, age 68 ± 9.2 years and BMI 31.1 ± 5.4 at inclusion) who participated in a two-year educational program for patients with KOA at the Osteometabolic Diseases Group Department of Orthopedics, Hospital das Clínicas, University of São Paulo participated in this study. 

The patients had to meet the following criteria: outpatients who were aged 45 years or older with KOA diagnosed according to the American College of Rheumatology clinical and radiological definition,^11^ had no rheumatoid arthritis or rheumatologic disease other than OA, had been receiving clinical treatment for OA in the past thirty months and had participated in the PARQVE educational program for patients with OAJ.^9^ ^,^ ^10^ The exclusion criteria were 1) KOA surgery during the study period or other surgery during the study period that would prevent regular physical activity, and 2) participation in another nutritional education program or another clinical study. Patients who were not able to perform or did not attend the functional tests were excluded only from the functional analysis.

### Intervention

At enrollment and 24 months later, the patients were asked to respond to VAS (visual analog pain scale), WOMAC(tm), Lequesne, and SF-36 questionnaires and to report the duration, intensity and type of physical activity they performed each week.^12^ ^-^ ^15^ The patients were asked to perform the timed up-and-go (TUG) test and the five times sit-to-stand (FTSST) test.^16^ ^-^ ^18^

All of the participants received information on OA disease and its treatment in the form of classes and/or educational materials that included the class content in text and video (DVD) form.^9^ ^,^ ^10^ The DVD was 2 hours and 23 minutes long. All of the patients were instructed to watch the DVD and/or read the handout at least three times and to exercise under the guidance of physical therapists or physical educators (via information of the handout or at public or private gyms) at least three times a week.

### Statistical analysis

Improvement criteria were established for each of the scores and functionality testing: a reduction of at least 10 points on the total WOMAC score, 4 points on the WOMAC pain score, 2 points on the WOMAC stiffness score, 5 points on the WOMAC physical function score, 4 seconds on the TUG and 4 seconds on the TSL. 

The quantitative characteristics were described as improvement for each criterion with the use of summary measures (mean, standard deviation, median, minimum and maximum) and were compared using Student's t or Mann-Whitney tests. Improvements in each criterion were described according to their qualitative characteristics and association using likelihood ratios or chi-square tests.

Odds ratios (ORs) were estimated for the association between each variable of interest and improvement in each criterion along with the respective 95% confidence intervals, based on simple logistic regression.

Multiple saturated logistic regression models were estimated for each of the improvement criteria.

The tests were performed at the 5% significance level.

## RESULTS

There was no relationship between improvement in the WOMAC, VAS, Lequesne, SF-36, TUG and FTSST scores and the type, intensity and duration of physical activity (all p> 0.05).

Table 1 shows that increasing age was associated with a lower likelihood of improvement in the TUG score, regardless of the other characteristics evaluated. With each additional year of age, there was a 7% reduction in the chance of improvement on the TUG test, OR = 0.93, p = 0.017.

**Table 1:** Description of quantitative characteristics according to improvements in the TUG time, qualitative characteristics and statistical test results.

Variable	Improves TUG		Total	OR unadjusted	IC (95%)		p	OR adjusted	IC (95%)		p
	No	Yes			Lower	Upper			Lower	Upper	
Type of physical activity, n (%)							0,561				
Sedentary	40 (85.1)	7 (14.9)	47	1.00				1.00			
Booklet/Stretching	26 (76.5)	8 (23.5)	34	1.76	0.57	5.43		#			0.999
Walking/Fitness/Water Aerobics	51 (87.9)	7 (12.1)	58	0.78	0.25	2.42		#			0.999
Swimming/Bicycle/Bodybuilding	17 (85)	3 (15)	20	1.01	0.23	4.37		#			0.999
Intensity of physical activity, n (%)							0.829				
Sedentary	44 (88)	6 (12)	50	1.00				1.00			
Light	54 (83.1)	11 (16.9)	65	1.49	0.51	4.36		#			0.999
Moderate	29 (85.3)	5 (14.7)	34	1.26	0.35	4.53		#			0.999
Vigorous	7 (77.8)	2 (22.2)	9	2.10	0.35	12.53		#			0.999
Minutes per Week				0.999	0.995	1.002	0.598*	0.995	0.988	1.002	0.176
Mean (SD)	124.7 (136.9)	101.7 (105.6)	121.2 (132.6)								
Median (min.; max.)	100 (0; 840)	70 (0; 390)	95 (0; 840)								
Age (years)				0.96	0.92	1.01	0.144	0.93	0.87	0.99	0.017
Mean (SD)	67.6 (8.5)	64.8 (10)	67.2 (8.8)								
Median (min.; max.)	68 (48; 87)	65 (47; 84)	67 (47; 87)								
Gender, n (%)							0.194*				
Male	32 (91.4)	3 (8.6)	35	1.00				1.00			
Female	103 (82.4)	22 (17.6)	125	2.28	0.64	8.11		4.89	0.55	43.66	0.155
Race, n (%)							0.536				
White	86 (83.5)	17 (16.5)	103	1.00				1.00			
Mulatto/Mestizzo	26 (81.2)	6 (18.8)	32	1.17	0.42	3.27		0.70	0.20	2.52	0.587
Black	18 (90)	2 (10)	20	0.56	0.12	2.65		0.35	0.06	2.02	0.24
Asian	4 (100)	0 (0)	4	#				#			0.999
Bilateral, n (%)							0.165*				
No	46 (90.2)	5 (9.8)	51	1.00				1.00			
Yes	89 (81.7)	20 (18.3)	109	2.07	0.73	5.86		2.36	0.71	7.87	0.163
BMI				1.04	0.96	1.13	0.356	0.98	0.87	1.12	0.799
Mean (SD)	31.2 (5.3)	32.3 (5.1)	31.4 (5.2)								
Median (min.; max.)	30.7 (19.8; 49.9)	31.2 (25.9; 45.8)	30.8 (19.8; 49.9)								
BFP				1.02	0.97	1.08	0.409	1.00	0.89	1.14	0.961
Mean (SD)	38.1 (8.4)	39.6 (7.7)	38.3 (8.3)								
Median (min.; max.)	40.4 (10.1; 49.1)	40.7 (18.5; 48)	40.4 (10.1; 49.1)								
Study time				0.99	0.86	1.14	0.721*	0.96	0.82	1.13	0.6
Mean (SD)	8 (3.1)	7.9 (3)	8 (3)								
Median (min.; max.)	8 (1; 15)	8 (1; 15)	8 (1; 15)								

The total number of cases varies by variable; Interaction between the type of activity and intensity (p> 0.999); Quantitative variables: Student's t test; * Mann-Whitney test; qualitative variables: the likelihood ratio test; * Chi-square test.

When comparing patients who engaged in intense physical activity with those who did not engage in any physical activity, we found that at enrollment, the few (three) patients who practiced intense physical activity had less pain than the 157 sedentary patients (p=0.02). After two years of participating in the PARQVE^9^ ^,^ ^10^ program, the 19 patients who practiced intense physical activity completed the TUG an average of 4 seconds faster than the sedentary group (p = 0.01, Table 2) and reported less pain (50.9±15.3) than those who did not engage in any physical activity (59.4±23.1) but not significantly (Table 2).

**Table 2:** Description of the test and scale results at baseline and after 2 years for patients who did not engage in regular physical activity and patients who engaged in intense regular physical activity and the results of comparative tests.

Variable		Baseline				p		2 years				p
		n	Sedentary	n	Vigorous activity			n	Sedentary	n	Vigorous activity	
WOMAC pain	Baseline					0.10	2 years					0.28
Mean (SD)	157	8.9 (4.2)	3	5 (1.7)	56	8.7 (3.9)	20	7.4 (3.4)
Median (min.; max.)		9 (0 - 20)		4 (4 - 7)		8 (1 - 17)		7 (2 - 15)
WOMAC stiffness					0.31					0.60
Mean (SD)	157	3.4 (1.9)	3	2.3 (1.2)		56	3.2 (2.1)	20	2.9 (2.0)	
Median (min.; max.)		3 (0 - 8)		3 (1 - 3)			3 (0 - 8)		3 (0 - 6)	
WOMAC function limitation					0.10					0.24
Mean (SD)	157	31.7 (12.4)	3	20.7 (8.1)		56	31 (14.0)	20	26.6 (12.8)	
Median (min.; max.)		32 (4 - 63)		16 (16 - 30)			32 (2 - 65)		27.5 (2 - 46)	
WOMAC total					0.10					0.26
Mean (SD)	157	44.5 (18.2)	3	28.7 (6.4)		56	42.8 (19.1)	20	36.8 (17.4)	
Median (min.; max.)		45 (5 - 87)		26 (24 - 36)			42 (0 - 84)		36 (6 - 64)	
VAS					0.02*					0.08
Mean (SD)	157	58.1 (26.4)	3	23 (3.5)		56	59.4 (23.1)	20	50.9 (15.3)	
Median (min.; max.)		60 (2 - 100)		25 (19 - 25)			60 (5 - 100)		51.5 (20 - 80)	
Lequesne					0.10					0.09
Mean (SD)	157	11.6 (4.2)	3	8.3 (2.1)		56	12.1 (4.9)	20	10.7 (3.8)	
Median (min.; max.)		12 (1.5 - 24)		9 (6 - 10)			12.3 (0 - 21)		10.8 (2.5 - 18.5)	
SF 36 - PCS					0.13					0.67
Mean (SD)	157	32.8 (8.2)	3	38.8 (4.3)		56	34.4 (9.2)	20	35.4 (7.6)	
Median (min.; max.)		32.1 (14.3 - 58.1)		21.2 (33.8 - 41.4)			34.4 (15.5 - 57.2)		33.1 (24.4 - 53.8)	
SF 36 - MCS					0.26					0.55
Mean (SD)	157	45.6 (12.6)	3	53.0 (8.6)		56	45 (13.2)	20	47.0 (11.8)	
Median (min.; max.)		44.5 (17.1 - 71.6)		51.2 (45.5 - 62.4)			44.5 (17.3 - 68.7)		43.7 (30.7 - 66.8)	
Timed Up and Go					0.99					0.01*
Mean (SD)	149	12.3 (4.0)	2	15.5 (11.7)		52	14.6 (11.6)	19	9.7 (2.1)	
Median (min.; max.)		11.2 (5.2 - 28.6)		15.5 (7.2 - 23.7)			11.3 (7.0 - 84.6)		9.4 (6.2 - 14.6)	
Five times sit to stand					0.14					0.34
Mean (SD)	140	23 (8.2)	2	15.6 (5.7)		49	22.1 (12.1)	19	18.6 (5.0)	
Median (min.; max.)			22 (11.1 - 66.4)		15.6 (11.6 - 19.6)				20.8 (10.5 - 81.6)		18.0 (10.5 - 27.8)	

After two years of the program, the 46 patients who exercised 180 minutes or more per week also completed the TUG an average of 4 seconds faster than the 56 sedentary patients (p<0.001, Table 3).

**Table 3:** Description of the 2-year scale and test results for patients who did not engage in regular physical activity and patients who exercised 180 minutes or more/week and the results of comparative tests.

Variable		Time of physical activity per week			p
	n	Sedentary	n	≥180 min. exercise	
WOMAC pain	60		48		0.28
Mean (SD)	8.7 (3.7)	7.8 (3.4)
Median (min.; max.)	8 (1 - 17)	2 (2 - 15)
WOMAC stiffness	60		48		0.79
Mean (SD)		3.4 (2.1)		3.4 (1.8)	
Median (min.; max.)		3 (0 - 8)		4 (0 - 6)	
WOMAC function limitation	60		48		0.35
Mean (SD)		30.9 (14.0)		28.0 (12.9)	
Median (min.; max.)		32 (2 - 65)		30 (2 - 51)	
WOMAC total	60		48		0.38
Mean (SD)		42.6 (18.4)		38.9 (17.3)	
Median (min.; max.)		42 (0 - 84)		41 (6 - 70)	
VAS	60		48		0.12
Mean (SD)		59.2 (22.7)		52.5 (21.8)	
Median (min.; max.)		60 (5 - 100)		58 (0 - 94)	
Lequesne	60		48		0.08
Mean (SD)		12.0 (4.9)		10.4 (4.2)	
Median (min.; max.)		12.3 (0 - 21)		11 (2 - 18.5)	
SF 36 - PCS	60		48		0.18
Mean (SD)		34.1 (8.9)		36 (8)	
Median (min.; max.)		34.1 (17 - 57.2)		36.8 (19.6 - 53.8)	
SF 36 - MCS	60		48		0.20
Mean (SD)		45.2 (13.0)		48.3 (11.6)	
Median (min.; max.)		44.5 (17.3 - 68.7)		48.8 (20.8 - 66.8)	
Timed Up and Go	56		46		<0.001*
Mean (SD)		14.6 (11.2)		10.1 (3.3)	
Median (min.; max.)		11.3 (7.0 - 84.6)		9.2 (6.2 - 24.9)	
Five times sit to stand	53		45		0.46
Mean (SD)		22 (11.8)		20.5 (8.5)	
Median (min.; max.)		20.1 (10.5 - 81.6)		18.1 (12.3 - 51.5)	

The participants who performed regular physical activity engaged in a wide variety of activities (exercises provided in the program handout, water aerobics, yoga, walking, weight training, swimming, stretching, tai chi chuan, bike riding, Pilates). Bodybuilders reported less pain (p = 0.009) and performed the TUG approximately 4 seconds faster than sedentary patients (p = 0.01, Table 4). The addition of the results for those who swam to the body builders' results maintained the difference from sedentary patients in terms of pain (p = 0.03) and TUG times (0.04, Table 5).

**Table 4:** Description of the 2-year scale and test results for patients who did not engage in regular physical activity and patients who swam or participated in bodybuilding and the results of comparative tests.

Variable		n	Sedentary	n	Swimming	p		n	Sedentary	n	Bodybuilding	p
WOMAC pain	Swimming					0.37	Bodybuilding					0.1
Mean (SD)	56	8.7 (3.9)	7	7.4 (1.9)	56	8.7 (3.9)	12	6.4 (4.1)
Median (min.; max.)		8 (1 - 17)		6 (6 - 11)		8 (1 - 17)		7 (2 - 15)
WOMAC stiffness					0.98					0.4
Mean (SD)	56	3.2 (2.1)	7	3.1 (1.7)		56	3.2 (2.1)	12	2.6 (2.2)	
Median (min.; max.)		3 (0 - 8)		4 (0 - 5)			3 (0 - 8)		2.5 (0 - 6)	
WOMAC function limitation					0.63					0.1
Mean (SD)	56	31 (14.0)	7	29.2 (5.3)		56	31 (14.0)	12	23.3 (14.6)	
Median (min.; max.)		32 (2 - 65)		27 (22 - 37)			32 (2 - 65)		25.5 (2 - 45)	
WOMAC total					0.70					0.12
Mean (SD)	56	42.8 (19.1)	7	40.1 (7)		56	42.8 (19.1)	12	32.4 (19.6)	
Median (min.; max.)		42 (0 - 84)		36 (34 - 52)			42 (0 - 84)		36 (6 - 64)	
VAS					0.84					0.009*
Mean (SD)	56	59.4 (23.1)	7	59.6 (16.5)		56	59.4 (23.1)	12	42.6 (18.5)	
Median (min.; max.)		60 (5 - 100)		59 (44 - 93)			60 (5 - 100)		43 (12 - 75)	
Lequesne					0.69					0.06
Mean (SD)	56	12.1 (4.9)	7	11.6 (2.0)		56	12.1 (4.9)	12	9.2 (4.5)	
Median (min.; max.)		12.3 (0 - 21)		11.5 (9 - 14)			12.3 (0 - 21)		9 (2.5 - 18.5)	
SF 36 - PCS					1					0.26
Mean (SD)	56	34.4 (9.2)	7	34.4 (6.4)		56	34.4 (9.2)	12	37.4 (8.3)	
Median (min.; max.)		34.4 (15.5 - 57.2)		31.9 (28 - 43.7)			34.4 (15.5 - 57.2)		39.4 (24.4 - 53.8)	
SF 36 - MCS					0.45					0.13
Mean (SD)	56	45 (13.2)	7	41.0 (5.8)		56	45 (13.2)	12	51.1 (11.6)	
Median (min.; max.)		44.5 (17.3 - 68.7)		42.5 (33 - 47.2)			44.5 (17.3 - 68.7)		50.4 (32 - 66.8)	
Timed Up and Go					0.86					0.01*
Mean (SD)	52	14.6 (11.6)	7	11.7 (3.1)		52	14.6 (11.6)	11	9.4 (2.8)	
Median (min.; max.)		11.3 (7.0 - 84.6)		11.3 (8.5 - 17.9)			11.3 (7.0 - 84.6)		9.2 (6.2 - 14.6)	
Five times sit to stand					0.37					0.14
Mean (SD)	49	22.1 (12.1)	7	22.1 (5.9)		49	22.1 (12.1)	11	17.7 (5.3)	
Median (min.; max.)			20.8 (10.5 - 81.6)		22.9 (14.9 - 30)				20.8 (10.5 - 81.6)		15.4 (10.5 - 27.8)	

**Table 5:** Description of the 2-year scale and test results for patients who did not engage in regular physical activity and patients who swam or participated in bodybuilding and the results of comparative tests.

Variable	n	Sedentary	n	Bodybuilding or swimming	p
WOMAC pain					0.12
Mean (SD)	56	8.7 (3.9)	19	6.9 (3.5)
Median (min.; max.)		8 (1 - 17)		7 (2 - 15)
WOMAC stiffness					0.54
Mean (SD)	56	3.2 (2.1)	19	2.8 (2.0)	
Median (min.; max.)		3 (0 - 8)		3 (0 - 6)	
WOMAC function limitation					0.13
Mean (SD)	56	31 (14.0)	19	25.5 (12.2)	
Median (min.; max.)		32 (2 - 65)		27.5 (2 - 45)	
WOMAC total					0.16
Mean (SD)	56	42.8 (19.1)	19	35.3 (16.3)	
Median (min.; max.)		42 (0 - 84)		36 (6 - 64)	
VAS					0.03*
Mean (SD)	56	59.4 (23.1)	19	48.9 (19.2)	
Median (min.; max.)		60 (5 - 100)		45 (12 - 93)	
Lequesne					0.10
Mean (SD)	56	12.1 (4.9)	19	10 (3.9)	
Median (min.; max.)		12.3 (0 - 21)		11 (2.5 - 18.5)	
SF 36 - PCS					0.39
Mean (SD)	56	34.4 (9.2)	19	36.3 (7.6)	
Median (min.; max.)		34.4 (15.5 - 57.2)		38.2 (24.4 - 53.8)	
SF 36 - MCS					0.47
Mean (SD)	56	45 (13.2)	19	47.4 (10.9)	
Median (min.; max.)		44.5 (17.3 - 68.7)		44.4 (32 - 66.8)	
Timed Up and Go					0.04*
Mean (SD)	52	14.6 (11.6)	18	10.3 (3.1)	
Median (min.; max.)		11.3 (7.0 - 84.6)		9.6 (6.16 - 17.9)	
Five times sit to stand					0.57
Mean (SD)	49	22.1 (12.1)	18	19.4 (5.8)	
Median (min.; max.)		20.8 (10.5 - 81.6)		17.7 (10.5 - 30)	

## DISCUSSION

As the most common form of joint disease, osteoarthritis (OA) is associated with an extremely high economic burden. This burden is largely attributable to the effects of disability, comorbid disease, and the expense of treatment.^19^

Among a cohort of 5,715 adults aged 65 years or older with arthritis, a lack of regular vigorous physical activity was the most prevalent risk factor (64%); it almost doubled the odds of functional decline (adjusted OR 1.9, 95% confidence interval 1.5-2.4) after controlling for all risk factors. If all subjects engaged in regular vigorous physical activity, the expected functional decline could be reduced by as much as 32%.^8^

In our series, we could not show any relation between improved pain, function or quality of life scores and the intensity, duration or type of physical activity (all p>0.05). This lack of association may be explained by the relatively small number of patients who engaged in intense physical activity (20) and by the fact that although many of our patients reported that they performed the exercises provided in the educational booklet on a regular basis, they did not actually do all of them (several chose to perform only the stretching exercises, and those who did perform the strength exercises did not regularly increase the load). However, age emerged as a barrier to improving inferior limb strength, function and balance, as determined by TUG performance^16^ ^,^ ^17^; this finding is consistent with those of Dunlop et al.^8^

The patients who already practiced intense physical activity at the time of enrollment reported less pain than those that were sedentary (Table 2). This finding is supported by a meta-analysis of 54 eligible trials (20 pharmacology, 34 exercise), including six Cochrane reviews (four pharmacology, two exercise,) with 9806 participants (5627 pharmacology, 4179 exercise). This meta-analysis reported a pooled effect size for pharmacological pain interventions of 0.41 (95% CI: 0.23-0.59); for exercise, the pooled effect size was 0.46 standardized mean difference (95% CI: 0.34-0.59). This meta-analysis provided indirect evidence that for KOA pain, the effects of exercise and those of oral analgesics are comparable.^20^

After two years, the group that engaged in intense physical activity performed the TUG test faster (9.7±2.1 seconds) than those who remained sedentary (14.6±11.6 seconds, p=0.01, Table 2), in accordance with the expected effect of intense physical activity described above.^8^

Not all patients are able to engage in intense physical activity (in our series, only 20 did so; some but not all of the other patients were capable of engaging in intense physical activity); in such cases, the weekly duration and type of physical activity may compensate for a decrease in intensity. Forty-six subjects engaged in regular physical activity for 180 minutes per week or more; on average, those patients performed the TUG test 4 seconds faster than the 56 patients who remained sedentary (p<0.001, Table 3). Regular exercise for 180 minutes or more per week may become goal or a means to performing intense physical activity in the future among patients with KOA.

The large variety of physical activity types was definitely responsible for the lack of significant correlations. The participants who participated in bodybuilding (weight training at the gym) and swimming, activities closely related to greater exercise intensity, reported less pain (p=0.009 and p=0.04, respectively) and faster TUG performances compared with the sedentary participants (p=0.001 and p=0.03, respectively, Tables 4 and 5).

Our study has limitations. It is a retrospective cohort study in which few participants engaged in regular intense physical activity and with considerable variety in the types of physical activities that the participants reported. Both, diversity of physical activity and number of participants actually engaging intense physical activity, diminished the power of the test for the N and demand prospective studies for this question. Among the study's strengths, it provides directions for future prospective randomized studies, such as carefully selecting the included age groups and restricting the types, duration and intensity of physical activity.

## CONCLUSION

Patients with KOA should aim for intense physical activity and/or more than 180 minutes of exercise per week and/or weight training (bodybuilding) for relevant pain reduction and functional improvement.
